# Morphological and Proteomic Analyses Reveal that Unsaturated Guluronate Oligosaccharide Modulates Multiple Functional Pathways in Murine Macrophage RAW264.7 Cells

**DOI:** 10.3390/md13041798

**Published:** 2015-03-30

**Authors:** Xu Xu, De-Cheng Bi, Chao Li, Wei-Shan Fang, Rui Zhou, Shui-Ming Li, Lian-Li Chi, Min Wan, Li-Ming Shen

**Affiliations:** 1 College of Life Science, Shenzhen Key Laboratory of Marine Bioresources and Ecology, Shenzhen University, Shenzhen 518060, China; E-Mails: xuxu@szu.edu.cn (X.X.); 215017325@qq.com (D.-C.B.); 611279860@qq.com (C.L.); 1210417703@qq.com (W.-S.F.); zhouruiswg@gmail.com (R.Z.); 2 College of Life Science, Shenzhen Key Laboratory of Microbial Genetic Engineering, Shenzhen University, Shenzhen 518060, China; E-Mail: msbiotools@gmail.com; 3 National Glycoengineering Research Center, Shandong University, Jinan 250100, China; E-Mail: lianlichi@gmail.com; 4 Division of Physiological Chemistry 2, Department of Medical Biochemistry and Biophysics, Karolinska Institute, Stockholm 17177, Sweden; E-Mail: min.wan@ki.se

**Keywords:** guluronate oligosaccharide, macrophage activation, nuclear factor-κB, anti-inflammation, antioxidant, cell morphology

## Abstract

Alginate is a natural polysaccharide extracted from various species of marine brown algae. Alginate-derived guluronate oligosaccharide (GOS) obtained by enzymatic depolymerization has various pharmacological functions. Previous studies have demonstrated that GOS can trigger the production of inducible nitric oxide synthase (iNOS)/nitric oxide (NO), reactive oxygen species (ROS) and tumor necrosis factor (TNF)-α by macrophages and that it is involved in the nuclear factor (NF)-κB and mitogen-activated protein (MAP) kinase signaling pathways. To expand upon the current knowledge regarding the molecular mechanisms associated with the GOS-induced immune response in macrophages, comparative proteomic analysis was employed together with two-dimensional electrophoresis (2-DE), matrix-assisted laser desorption/ionization time-of-flight mass spectrometry (MALDI-TOF/TOF MS) and Western blot verification. Proteins showing significant differences in expression in GOS-treated cells were categorized into multiple functional pathways, including the NF-κB signaling pathway and pathways involved in inflammation, antioxidant activity, glycolysis, cytoskeletal processes and translational elongation. Moreover, GOS-stimulated changes in the morphologies and actin cytoskeleton organization of RAW264.7 cells were also investigated as possible adaptations to GOS. This study is the first to reveal GOS as a promising agent that can modulate the proper balance between the pro- and anti-inflammatory immune responses, and it provides new insights into pharmaceutical applications of polysaccharides.

## 1. Introduction

Alginate is currently extracted from marine brown algae and is known to be arranged in homopolymeric α-l-guluronate (G) blocks (polyguluronate, PG), β-d-mannuronate blocks (M) (polymannuronate, PM) and random heteropolymeric G and M stretches [1]. Alginate is used for biotechnological and medical purposes in a wide range of commercial applications in the pharmaceutical field. Alginate oligosaccharide (AOS), which is obtained by lyase depolymerization of polymer and has a relatively low molecular weight, is regarded as a non-toxic, biocompatible, nonimmunogenic and biodegradable polymer, making it an attractive candidate for biomedical applications [2]. This oligosaccharide has various physiological functions, such as the promotion of bifidobacterial growth [3], the stimulation of endothelial cell growth and migration [4], and human keratinocyte growth [5]. AOS is also involved in the induction of cytokine production in macrophages [6,7,8], the enhancement of protection against infections by certain pathogens [9], antioxidant [10,11] and neuroprotective activities [12], and the suppression of Th2 development and IgE secretion through the induction of IL-12 secretion [13].

Macrophages play major roles in host defense, immunity and inflammatory responses and help to maintain steady-state tissue homeostasis [14]. The inflammatory response is a self-limiting process and involves the sequential activation of signaling pathways leading to the production of both pro- and anti-inflammatory mediators [15]. Our recent studies have demonstrated that enzymatically depolymerized guluronate oligosaccharide (GOS) from PG could markedly increase phagocytosis of IgG-opsonized *Escherichia coli* and *Staphylococcus aureus* and intracellular bacterial killing by macrophages, resulting in enhancement of the antibacterial activity of macrophages via the activation of several signaling pathways that are related to innate immunity and bacterial clearance in murine acute peritonitis *in vivo* [16]. GOS activates macrophages by binding to Toll-like receptor (TLR) 2/4, causing cytokine production [17]. We have also confirmed that GOS activates the nuclear factor (NF)-κB and mitogen-activated protein (MAP) kinase signaling pathways, elevates inducible nitric oxide synthase (iNOS) expression and, in turn, nitric oxide (NO) production, and induces tumor necrosis factor (TNF)-α secretion and reactive oxygen species (ROS) production [18]. GOS stimulates cellular inflammatory responses and releases factors, which indicates that it could be used in the agriculture, food and drug industries as a potent immunomodulatory agent.

Proteomics is now generally accepted as a useful method due to its high-throughput capability to analyze total protein expression and elucidate cellular processes at the molecular level [19,20]. To gain more information and elucidate specific mechanisms underlying the involvement of GOS involved in phagocytosis and signal transduction pathways, we carried out a proteomic study to identify differentially expressed proteins extracted from control and GOS-treated RAW264.7 cells using two-dimensional electrophoresis (2-DE) and matrix-assisted laser desorption/ionization time-of-flight mass spectrometry (MALDI-TOF/TOF MS). We identified nine proteins with significant changes (fold change ≥ 2.0) in expression between the GOS-treated and untreated RAW264.7 cells. These results were confirmed by Western blot, biochemical studies and morphological analyses. Considered together with the findings of our previous studies [16,18], the current results suggest that the functional pathways of activation and negative regulation of the NF-κB signaling pathway, pro- and anti-inflammation, pro- and anti-oxidation, cytoskeletal remodeling and cell proliferation might be involved in GOS-induced macrophage activation and immunomodulation. Notably, the inflammatory response requires the coordinated activation of various signaling pathways that regulate the expression of both pro- and anti-inflammatory mediators [15]. Thus, we provide key insight into the potential mechanisms by which the immune response is modulated by GOS.

## 2. Results

### 2.1. Preparation and Structural Analysis of GOS

**Figure 1:**
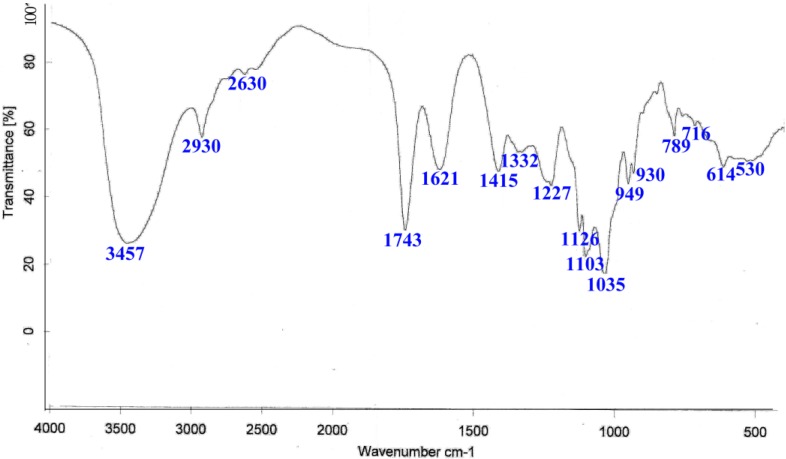
The infrared (IR) spectrum of polyguluronic acid (PG). The spectrum was run in KBr pellets with 1 mg of sample and 200 mg of KBr.

As shown in Figure 1, IR spectroscopy revealed characteristic peaks at 3457, 2930, 1743, 1621, 1415, 1126, 1103, 1035 and 789 cm^−1^. The broad absorption band at 3457 cm^−1^ is representative of the stretching frequency of OH groups, and the band at 2930 cm^−1^ is attributable to CH asymmetric stretching. The weak absorption peak at 1743 cm^−1^ indicates the presence of uronic acids, and the absorption peaks at 1621 cm^−1^ and 1415 cm^−1^ are also representative of polysaccharides. The region at 1200–1000 cm^−1^ contained three absorption peaks indicative of a pyranoid saccharide [21]. The very weak absorption peak at 789 cm^−1^ is a unique characteristic of guluronic acid residues.

**Figure 2:**
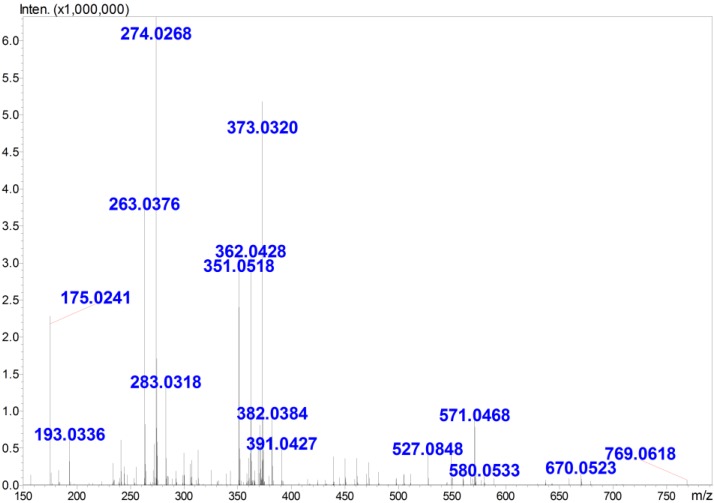
The electrospray ionization mass spectrometry (ESI-MS) of guluronate oligosaccharide (GOS). The spectrum was acquired in the negative ion mode with a high-resolution hybrid time-of-flight mass spectrometer. The ions were present in the form of [M + *x*Na(K) − (*x* + *n*)H]^*n*−^. The corresponding molecular weights and DP were then calculated using the monoisotopic peaks and charge states of each group of ions.

Furthermore, the molecular weight and degree of polymerization (DP) of GOS, which was enzymatically digested from PG, was determined using ESI-MS. The results indicated (Figure 2) that multiple charged ions, which were associated with different amounts of sodium or potassium adducts, were observed for GOS in the negative mode ESI-MS analysis. The charge states of the ions were deduced based on their isotopic distributions, and their molecular weights were then calculated. The interpretation of the MS peaks (Table 1) revealed that the primary GOS ranged from dimers to octamers (G2–G8).

**Table 1:** Ions observed in the mass spectrometry (MS) analysis of GOS.

*m/z*	Charge State	Ion Format	Corresponding DP ^(a)^	MW ^(b)^
351	1	[M − H]^−^	2	352
263	2	[M − 2H]^2−^	3	528
274	2	[M + Na − 3H]^2−^	3	528
283	2	[M + K − 3H]^2−^	3	528
571	1	[M + 2Na − 3H]^−^	3	528
362	2	[M + Na − 3H]^2−^	4	704
373	2	[M + 2Na − 4H]^2−^	4	704
382	2	[M + 2Na − 4H + H_2_O]^2−^	4	704
439	2	[M − 2H]^2−^	5	880
450	2	[M + Na − 3H]^2−^	5	880
461	2	[M + 2Na − 4H]^2−^	5	880
472	2	[M + 3Na − 5H]^2−^	5	880
481	2	[M + 3Na-5H + H_2_O]^2−^	5	880
527	2	[M − 2H]^2−^	6	1056
538	2	[M + Na − 3H]^2−^	6	1056
549	2	[M + 2Na − 4H]^2−^	6	1056
560	2	[M + 3Na − 5H]^2−^	6	1056
571	2	[M + 4Na − 6H]^2−^	6	1056
670	2	[M + 5Na − 7H]^2−^	7	1232
679	2	[M + 5Na − 7H + H_2_O]^2−^	7	1232
490	3	[M + 3Na − 6H]^3−^	8	1407
497	3	[M + 4Na − 7H]^3−^	8	1406

^(a)^ DP = degree of polymerization; ^(b)^ MW = molecular weight.

### 2.2. Comparison of Protein Expression Patterns between GOS-Treated and Control Cells

**Figure 3:**
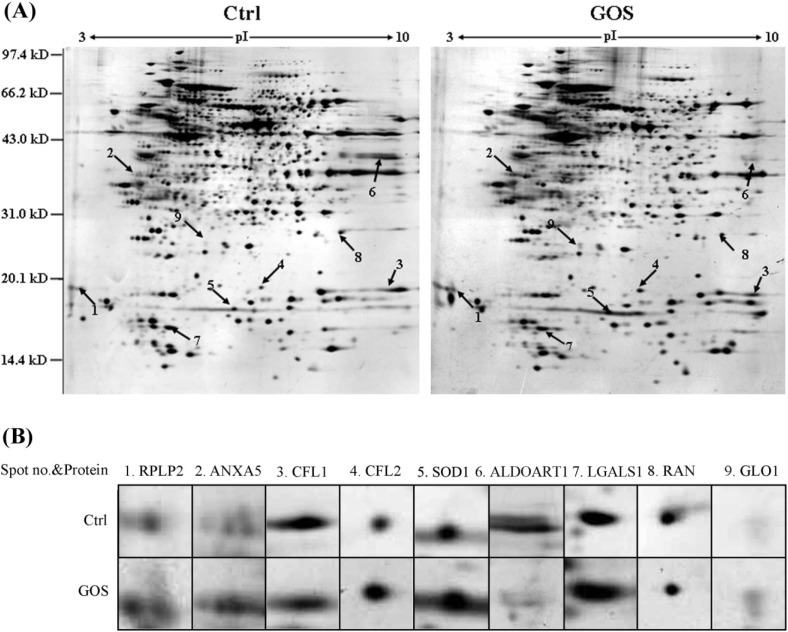
(**A**) A representative two-dimensional electrophoresis (2-DE) gel showing of total proteins from untreated and GOS-treated RAW264.7 cells. Each gel is representative of three independent replicates. Differentially regulated proteins (≥2.0-fold) are indicated by arrows and numbers and are listed in Table 2; (**B**) Magnified image of an identified protein spot.

To explore the underlying mechanisms of the effects of GOS on RAW264.7 cells, comparative proteomic analyses were performed. After RAW264.7 cells were incubated with 1 mg/mL GOS in FBS-free culture medium for 24 h, proteins were extracted and separated by 2-DE. Representative silver-stained 2-DE maps are shown in Figure 3. A comparison of these maps with the images revealed that the expression of nine proteins was significantly altered (by ≥2.0-fold), and these proteins were identified by MALDI-TOF/TOF MS analysis, as shown in Table 2. Among them, six proteins were significantly up-regulated, and three were noticeably down-regulated. The up-regulated proteins were identified as 60S acidic ribosomal protein P2 (RPLP2), annexin A5 (ANXA5), cofilin-2 (CFL2), Cu/Zn-superoxide dismutase (SOD1), galectin-1 (LGALS1) and lactoylglutathione lyase (GLO1). The down-regulated proteins were cofilin-1 (CFL1), fructose-bisphosphate aldolase (ALDOART1) and GTP-binding nuclear protein (RAN).

The proteins could be divided into six functional categories according to the SwissProt database. The first group was related to the NF-κB signaling pathway and included LGALS1 and GLO1. The second group comprising ANXA5 and RAN was related to inflammation. The third group was related to oxidative stress and included SOD1. The fourth group was related to metabolic processes and included ALDOART1. The fifth group, including CFL1 and CFL2, was involved in cytoskeletal processes. The sixth group was related to translational elongation and included RPLP2.

**Table 2:** Detailed information regarding the differentially expressed proteins detected by MS.

Spot No.	Protein ID	Symbol	Accession Number	MW (kD)/pI	Peptides Matched ^(a)^	Cov (%) ^(b)^	Protein Score	Expr Level ^(c)^	Reported Function
1	60S acidic ribosomal protein P2	RPLP2	P99027	11.65/4.38	3(2)	31	168	+3.3 ± 0.3	Translation
2	Annexin A5	ANXA5	P48036	35.75/4.82	7(6)	30	244	+2.7 ± 0.2	Inflammation
3	Cofilin-1	CFL1	P18760	18.56/8.22	4(3)	26	256	−2.1 ± 0.2	Cell cytoskeleton
4	Cofilin-2	CFL2	P45591	18.71/7.66	1(1)	6	73	+2.4 ± 0.2	Cell cytoskeleton
5	Cu/Zn-superoxide dismutase	SOD1	P08228	15.94/6.02	4(4)	24	315	+2.1 ± 0.2	Antioxidant
6	Fructose-bisphosphate aldolase	ALDOART1	Q9CPQ9	39.35/8.30	5(4)	18	296	−5.9 ± 0.7	Metabolic process
7	Galectin-1	LGALS1	P16045	14.86/5.28	6(6)	43	426	+2.3 ± 0.3	Signal transduction
8	GTP-binding nuclear protein	RAN	P62826	24.42/7.01	6(5)	21	324	−2.2 ± 0.2	Inflammation
9	Lactoylglutathione lyase	GLO1	Q9CPU0	20.81/5.24	2(2)	8	65	+2.4 ± 0.2	Signal transduction

^(a)^ Peptides matched by mass fingerprinting; ^(b)^ Protein sequence coverage; ^(c)^ Expression level in GOS-treated RAW264.7 cells at 24 h compared with control cells (+, increase; −, decrease).

### 2.3. Western Blot Analysis for Validation of Differentially Expressed Proteins

**Figure 4:**
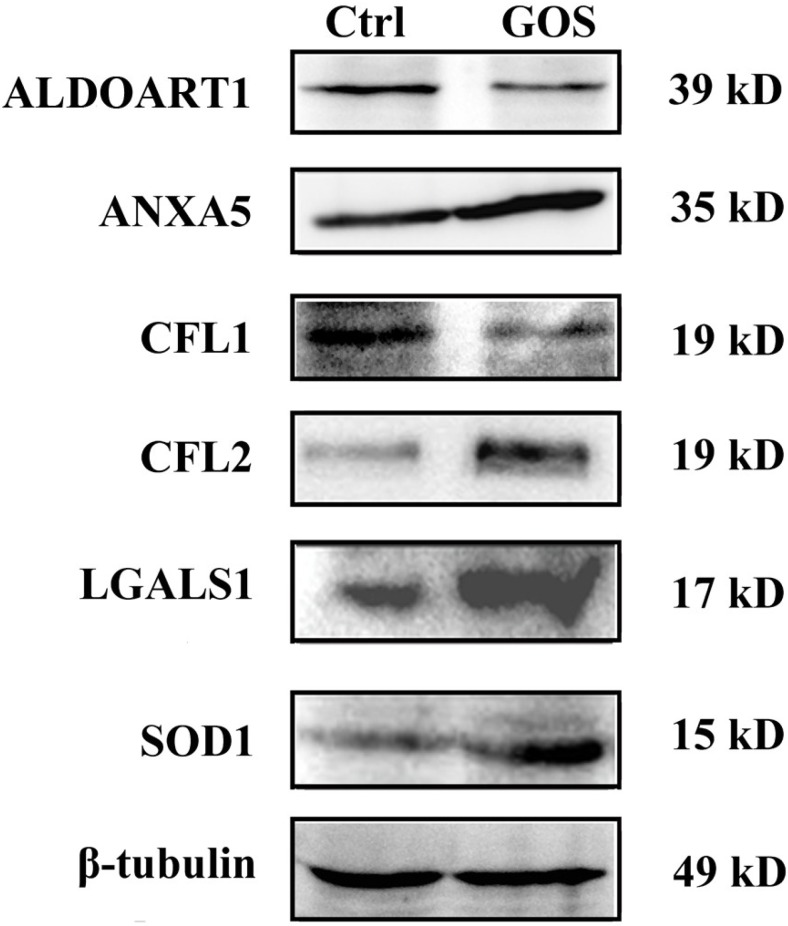
Western blot analysis of altered proteins in GOS-treated RAW264.7 cells. A representative result of three independent experiments is shown. Typical experiment conducted three times with similar results. β-tubulin was used as an internal control.

Western blot analysis was carried out to confirm the differential expression of ALDOART1, ANXA5, CFL1, CFL2, LGALS1 and SOD1. Figure 4 shows that the expression of ANXA5, CFL2, LGALS1 and SOD1 was significantly up-regulated, whereas the expression of ALDOART1 and CFL1 was clearly down-regulated in GOS-stimulated RAW264.7 cells compared with control cells, which corresponded well with the differences observed in 2-DE analysis.

### 2.4. Effects of GOS on the Morphology and Actin Cytoskeleton Organization of RAW264.7 Cells

**Figure 5:**
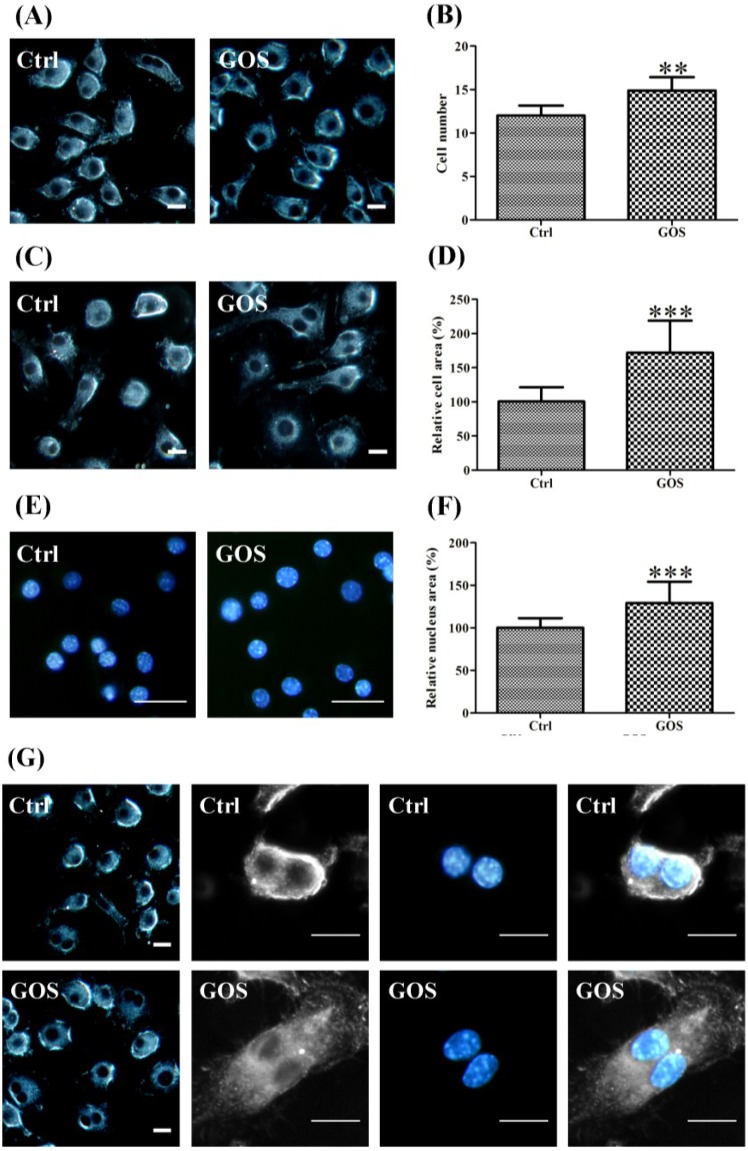

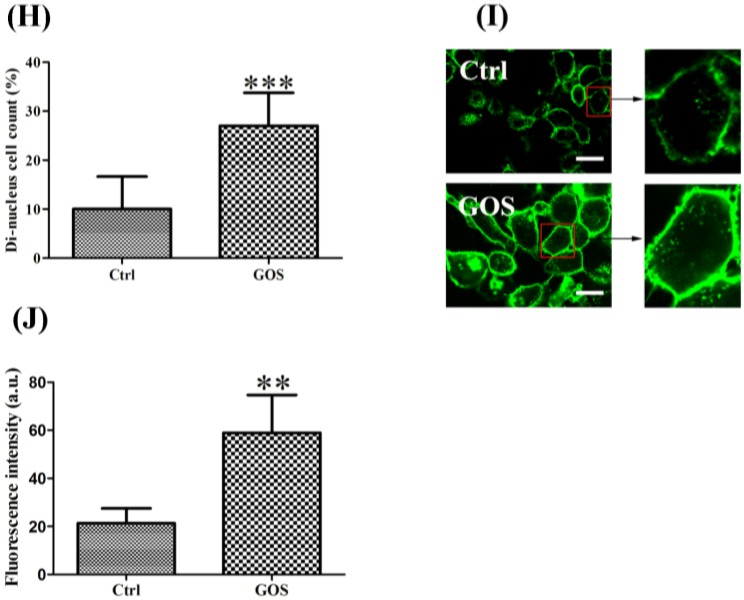
Effects of GOS on the morphology and actin organization of RAW264.7 cells. RAW264.7 cell morphology was observed by dark-field and confocal microscopy (×40). The cells were treated with or without 1 mg/mL GOS for 24 h. Representative dark-field images and analysis results show that GOS induced morphological changes in RAW264.7 cells, including an increase in the cell number (**A**,**B**), larger cell sizes (**C**,**D**), extended nucleus areas (**E**,**F**), and a greater number of dual-nuclei cells (**G**,**H**), compared with untreated cells (control). F-actin was stained with FITC-phalloidin. GOS-induced F-actin organization was examined using fluorescence images (**I**) and quantitative analysis (**J**). Scale bar, 20 µm. All images were analyzed with ImageJ software. ** *p* < 0.01 and *** *p* < 0.001 indicate significant differences between the control group and the GOS-treated group.

We observed changes in the morphology and actin cytoskeleton organization of GOS-treated RAW264.7 cells compared with control cells under dark-field and confocal microscopy (×40). Figure 5 shows that the GOS treatment induced morphological alterations in the macrophages, including a dramatic increase in cell number, cell size and nucleus size; an increased number of cells with two nuclei; and increased F-actin accumulation compared with untreated cells. As shown in Figure 5A, GOS had an obvious growth-promoting effect on RAW264.7 cells, which was reflected by a marked increase in the total number of cells. Quantitative analysis revealed that GOS treatment caused a 1.25-fold increase in the number of RAW264.7 cells compared with no treatment (Figure 5B). The majority of untreated RAW264.7 cells exhibited a rounded morphology. Treatment with GOS for 24 h stimulated the production of numerous hair-like membrane protrusions or filopodia, which led to an increase in the macrophage cell area, resulting in extended cellular spreading (Figure 5C). The relative cell size and relative nucleus area were measured using ImageJ software. By analyzing the relative cell size distribution of control and GOS-treated RAW264.7 cells, we found that GOS moderately increased the mean cell area to 172.0% ± 6.6% of the control (100%) (Figure 5D). The GOS-treated RAW264.7 cells also possessed larger nuclei and prominent nucleoli (Figure 5E), and the nuclear areas of the cells treated with GOS were 29.3% ± 3.5% larger than those of the untreated cells (100%) (Figure 5F). We propose a possible positive correlation between cellular uptake during phagocytosis in macrophages and increases in cell number, cell size and nucleus size in GOS-treated RAW264.7 cells. Furthermore, it is interesting to note that the GOS-treated RAW264.7 cells showed an increase in the number of nuclei (Figure 5G), and quantitative analysis revealed that GOS treatment caused a 2.7-fold increase in the number of cells with dual nuclei compared with the control (no treatment) (Figure 5H). Hence, it appears that GOS facilitates the formation of dual-nuclei cells.

In addition, to determine the cytoskeletal changes in RAW264.7 cells, F-actin was visualized with FITC-phalloidin staining. GOS-treated RAW264.7 cells showed an increase in F-actin expression (Figure 5I). Fluorescence intensity was quantified by ImageJ software. Figure 5J shows that GOS treatment induced a 2.8-fold increase in F-actin fluorescence intensity compared with untreated cells. These results support those of our previous study, showing that cytoskeletal transformation can increase the phagocytosis of bacteria by RAW264.7 cells [16].

### 2.5. Effects of GOS on Lipopolysaccharide-Activated Morphological Changes in RAW264.7 Cells

**Figure 6:**
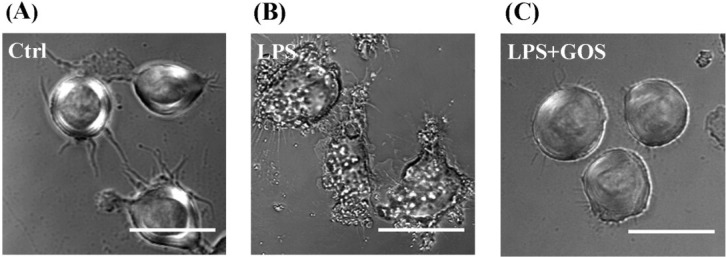
Effects of GOS on the lipopolysaccharide (LPS)-activated morphological changes in RAW264.7 cells. The morphologies of RAW264.7 cells were visualized with a phase contrast microscope (×40). The cells were pretreated with 1 mg/mL GOS for 2 h before incubation with 1 μg/mL LPS for 24 h. (**A**) Control; (**B**) LPS-treated only; and (**C**) LPS-treated with GOS. Scale bar, 20 µm.

Lipopolysaccharide (LPS), a macrophage activator, is known to rapidly activate morphological changes in macrophages. It has been reported that anti-inflammatory agents can generally inhibit the LPS-stimulated cell morphological changes [22,23]. To evaluate the effects of GOS on the morphologies of LPS-stimulated RAW264.7 cells, the cells were monitored under a phase contrast microscope (×40). The normal cells (control) were generally round and smooth with limited cell spreading and finely granulated cytoplasm, whereas the LPS-activated RAW264.7 cells displayed a significantly irregular and rough form with accelerated spreading and the formation of pseudopodia, relatively prominent cytoplasm with increased granularity, and condensed chromatin in the nucleus (Figure 6). Following pretreatment with GOS for 2 h and subsequent stimulation with LPS, the cells became rounded, and the levels of cell spreading and pseudopodia formation were reduced as expected. These results demonstrated that pretreatment with GOS could reduce LPS-stimulated irregular cell morphology, suggesting that GOS might specifically affect macrophage functions.

## 3. Discussion

In recent years, many reports have focused on the immunomodulatory effects of AOS [10,18,24]. GOS, which is derived from marine brown seaweed is generally non-toxic and shows a diverse range of beneficial biological activities. Additionally, GOS is regarded as a potential immunotherapeutic agent for the regulation of immune responses in the pharmacological industries [18]. Our previous study indicated that GOS activates the NF-κB signaling pathway, which modulates the expression of various genes involved in immune and inflammatory responses. For example, this pathway stimulates the production of NO, ROS, and TNF-α in macrophages [18]. To further determine the possible underlying biochemical mechanisms associated with GOS-induced immune response in macrophages, we identified differentially expressed proteins using 2-DE. Nine proteins were found to be significantly differentially expressed in GOS-treated cells compared with control cells, and these proteins were considered to be involved in the following processes: signal transduction; inflammatory reactions; antioxidant, metabolic, and cytoskeletal processes; and translational elongation. Further, the results of 2-DE proteomic analysis were validated by Western blot analysis.

The levels of LGALS1 and GLO1, two proteins that participate in signal transduction and are involved in the NF-κB signaling pathway, were increased following GOS treatment. Activation of NF-κB is critical for host defense [25]. However, NF-κB-driven immune responses must not be permanent; they need to be down-regulated and properly terminated [26]. LGALS1, a β-galactoside-binding protein belonging to the galectin family, has been reported to act as an endogenous potent anti-inflammatory factor and form a feedback regulatory loop with NF-κB [27]. NF-κB-activating stimuli increase LGALS1 expression in T cells; however, up-regulation of LGALS1 can inhibit the NF-κB signaling pathway [27,28,29]. We have reported that GOS activates NF-κB signaling pathway [16,18]. Thus, it could be speculated that GOS promotes NF-κB expression, thereby up-regulating LGALS expression and contributing to the attenuation of NF-κB overactivation. Another protein, GLO1, is involved in regulation of the TNF-induced transcriptional activity of NF-κB. Overexpression of GLO1 contributes to suppress TNF-induced NF-κB-dependent reporter gene expression, whereas a GLO1 specific knockdown significantly increases TNF-induced NF-κB expression [30]. Our results hinted that elevated levels of GLO1 might decrease the transcriptional activities of TNF-induced NF-κB-regulated target genes in GOS-treated RAW264.7 cells. Taken together, the up-regulation of LGALS1 and GLO1 inhibited the overactivation of the NF-κB signaling pathway in GOS-treated cells, which could be considered to be a negative feedback regulatory loop for the maintenance of cell homeostasis. In addition, LGALS1 has been shown to influence Fc gamma receptor (FcγR) expression and FcγR-dependent functions, such as phagocytosis, through active extracellular signal-regulated kinase (ERK)1/2-dependent pathway [31]. Interestingly, our previous reports revealed that GOS treatment enhanced the expression of FcγR II and FcγR III and activated the ERK MAP kinase signaling pathway in macrophages [16,18]. Hence, LGALS1 may play a role in the GOS-induced macrophage phagocytic activity.

ANXA5, a cytosolic Ca^2+^-binding protein, and RAN, a GTPase-activating protein, play important roles during the immune and inflammatory response [32,33,34]. ANXA5 decreases the anti-inflammatory cytokines, and ANXA5-knockout animals display increased anti-inflammatory and immunosuppressive potential [34]. RAN appears to be involved in the response to LPS [33], and a high level of RAN overexpression in both macrophages and B cells leads to the down-regulation of LPS signal transduction [35]. In this study, ANXA5 was dramatically up-regulated and RAN was down-regulated in GOS-treated RAW264.7 cells (Figure 4), suggesting that these proteins might contribute to GOS-induced pro-inflammatory effects.

SOD1 is known to be a specific scavenger of the superoxide anion. It is one of the major antioxidant enzymes in mammalian cells and is important for maintaining the balance between O_2_^·−^ generation and removal [36]. The production of ROS and reactive nitrogen species (RNS) (e.g., H_2_O_2_, HO^·^, O_2_^·−^ and NO) by immunocytes is considered to be essential in the destruction of invading pathogens [37]; however, radicals can also cause oxidative stress damage to the host cells. To protect themselves against constant oxidative challenges, cells develop defense mechanisms that ensure a proper balance between pro- and anti-oxidant actions [38]. GOS was able to augment ROS production [18] and up-regulate SOD1 expression in RAW264.7 cells (Figure 4), demonstrating that the activation of SOD1 can protect cells from oxygen radical overaccumulation. Moreover, recent reports have demonstrated that alginate and AOS have potent antioxidant abilities [10,11] and that AOS protects PC12 pheochromocytoma cells against H_2_O_2_-induced oxidative stress via the activation of antioxidant enzymes, including SOD [12]. Therefore, the increase in SOD1 activity in the GOS-treated cells might also be related to the antioxidant ability of alginate. In addition, the activation of macrophages leads to the secretion of SOD1 via ERK activation, resulting in increased release of TNF-α and the production of ROS, and the overexpression of SOD1 stimulates immune responses, including ROS production and further TNF-α secretion [39].

Oxidative stress, such as that caused by ROS overproduction and the activation of key transcription factors such as NF-κB, can promote aerobic glycolysis and inflammation [40]. It has been reported that glycolysis promotes the proinflammatory activation of macrophages [41], and inflammation can be prevented in mice by blocking the glycolytic metabolic pathway of macrophages [42]. ALDOART1 is a ubiquitous enzyme essential for glycolysis and gluconeogenesis [43]. ALDOART1 was down-regulated in GOS-treated RAW264.7 cells compared with control cells (Figure 4), which may have led to the inhibition of glycolysis and may have impacted cellular metabolism. This result suggests that ALDOART1 may be involved in the negative regulation of GOS-induced pro-inflammatory responses.

Morphological changes constantly occur in macrophages when quiescently surveying environment or after phagocytosis activation. The morphofunctional alterations of cells require active actin cytoskeletal remodeling and metabolic adaptation [14]. In macrophages and dendritic cells, the actin cytoskeleton has been shown to regulate chemotaxis, phagocytosis and antigen presentation [44]. The actin-depolymerizing factor (ADF)/cofilin family of actin binding proteins, which consists of CFL1 (non-muscle cofilin), CFL2 (muscle cofilin) and ADF, are essential regulators of actin filament turnover [45]. CFL1 promotes cytoskeletal dynamics by depolymerizing actin filaments, whereas CFL2 exhibits weaker actin filament depolymerization activity compared to CFL1 and promotes filament assembly [46,47,48]. Our results showed that GOS induced down-regulation of CFL1 expression and significant up-regulation of CFL2 expression in RAW264.7 cells (Figure 4), implying that GOS elicited synergistic effects on the inhibition of F-actin disassembly and the promotion of actin polymerization. Furthermore, it has been demonstrated that CFL1 is important for cell division, and its inactivation leads to abnormal F-actin accumulation and enhances macrophage phagocytic activity [44]. The depletion of CFL1 results in increases in the cell sizes of different cell types, including macrophages [47], and CFL1-knockdown macrophages contain two or more nuclei [44]. These findings prompted us to investigate the effects of GOS on the morphologies and actin cytoskeleton remodeling of RAW264.7 cells. As expected, after treatment with GOS, the accumulation of F-actin and morphological changes, such as increases in cell size, nucleus area and dual-nuclei cell number, were observed in the RAW264.7 cells. These morphological changes enabled the cells to dynamically adapt to particular stimuli, for example, by enhancing bacterial phagocytosis by GOS-treated RAW264.7 cells, as has been described in our previous report [16]. Moreover, GOS-treated RAW264.7 cells contained more dual-nuclei cells compared with untreated cells, demonstrating that CFL1 does not affect chromosome replication but that it may play an important role in cytokinesis, consistent with a previous report [47].

Furthermore, the total cell number was increased after treatment with GOS, suggesting that it may also promote the proliferation of RAW264.7 cells. Kawada *et al.* have reported that AOS enhances the growth of human endothelial cells and keratinocytes [4,5]. RPLP2 plays an important role in the elongation step of protein synthesis, and enhancement/reduction of RPLP2 expression can affect the rate of protein translation, thereby increasing/decreasing the proliferation rates of cells [49]. Here, we found that GOS stimulated an increase in RPLP2 expression (Figure 4), indicating that it may be responsible for GOS-induced cell proliferation.

It is worth noting that LPS, which is a well-known macrophage activator, is known to inhibit proliferation, induce apoptosis [50], and stimulate macrophage morphological changes [22,23]. LPS-induced apoptosis in macrophages results from two independent mechanisms: first and predominantly, this activity occurs through the autocrine secretion of TNF-α (early apoptotic events), and second, it occurs through the production of NO (late phase of apoptosis) [51]. Although GOS was able to induce TNF-α secretion and NO production [18], apoptotic-like changes were not observed in the GOS-treated macrophages. Furthermore, GOS appeared to protect RAW264.7 cells from cell morphological changes caused by LPS treatment. These findings suggest that different mechanisms or functional pathways might be implicated in the macrophage-activating activities of GOS and LPS. In addition, LPS directed the core metabolism of these cells toward aerobic glycolysis [52], whereas the glycolytic pathway appeared to be down-regulated by GOS in the present study. Furthermore, the changes in cell morphology were different between the LPS- and GOS-treated cells. Therefore, our study indicated that GOS was able to activate macrophages [18], but it could also control the over-inflammatory reaction and cell homeostasis; thus, its function was different from the function of LPS.

## 4. Materials and Methods

### 4.1. Materials

Seaweed sodium alginate (20 cps), bacterial lipopolysaccharide (LPS), dithiothreitol (DTT), iodoacetamide (IAA), sodium dodecyl sulfate (SDS) and polyacrylamide were purchased from Sigma-Aldrich (St. Louis, MO, USA). 3-[(3-cholamidopropyl) dimethylammonio]-1-propanesulfonate (CHAPS), Tris base, thiourea and urea were purchased from Amresco (Solon, OH, USA). Immobiline dry strips, immobilized pH gradient (IPG) buffer and IPG cover mineral oil were obtained from GE Healthcare (Fairfield, CT, USA). All reagents for silver-staining were purchased from Guangzhou Chemical Reagent Factory (Guangzhou, China). RPMI 1640 culture medium, fetal bovine serum, penicillin and streptomycin were obtained from Hyclone (Logan, UT, USA).

### 4.2. Preparation of GOS

PG (DP = 20-24) was prepared from sodium alginate according to Haug *et al.* [53], and its chemical structure was analyzed using a Nicolet 6700 infrared (IR) spectrophotometer (ThermoScientific, Rochester, NY, USA) according to Linker *et al.* [54]. Unsaturated GOS was depolymerized from PG by purified alginate lyase [55]. Before use, GOS was filtered through an endotoxin-removing filter (0.22 µm) (Millipore Co., Billerica, MA, USA).

### 4.3. Mass Spectrometry Analysis

Electrospray ionization mass spectrometry (ESI-MS) analysis was performed using an ion trap time-of-flight mass spectrometer (Shimadzu, Tokyo, Japan) in the negative mode. GOS was dissolved in 1 mM NH_3_·H_2_O in 50% aqueous methanol solution and diluted to a final concentration of 1 mg/mL. The sample was infused into the mass spectrometer using a built-in syringe pump at a flow rate of 10 µL/min. The instrument parameters were set as follows: an interface voltage of −3.0 kV; a nebulizing gas flow rate of 0.5 L/min; a CDL temperature of 200 °C; a heating block temperature of 200 °C; a detector voltage of 1.75 kV; and a scan range of 200–2000.

### 4.4. Cell Culture

The murine macrophage-like cell line RAW264.7 was obtained from the Shanghai Institute of Biochemistry and Cell Biology (Shanghai, China). RAW264.7 cells were cultured in RPMI 1640 medium plus 10% fetal bovine serum, 100 units/mL penicillin and 100 μg/mL streptomycin. The cells were maintained in a humidified incubator with an atmosphere of 95% air and 5% CO_2_ at 37 °C.

### 4.5. Protein Extraction and 2-DE

RAW264.7 cells were harvested and lysed in lysis buffer (7 M urea, 2 M thiourea, 4% CHAPS, 2% pharmalyte (pH 3–10), 65 mM DTT and 40 mM Tris base), sonicated 10 times each for 5 s each with a 10-s pause between pulses in an ice-water bath and centrifuged at 14,000 rpm for 60 min at 4 °C. The supernatant was used directly for 2-DE analysis. Protein concentrations were determined using the Bradford assay.

Whole cell protein lysates were loaded onto analytical gels (90 μg) and MS-preparative gels (500 μg), and 2-DE was performed as described previously [56]. Briefly, IPG strips were rehydrated for 12 h at 30 V. Isoelectric focusing (IEF) was performed with the following voltage program: 100 V/2 h, 200 V/1 h, 500 V/1 h, linear ramp to 1000 V over 1 h, 8000 V over 3 h, then 8000 V constant for a total focusing time of 50,000 Vh. After IEF, the proteins were reduced and alkylated. The second dimension was carried out using an SE 600 Ruby system (GE Healthcare, Fairfield, CT, USA) by SDS-PAGE with 12.5% polyacrylamide gels. After migration, silver nitrate and Coomassie brilliant blue (CBB) R-250 were used to stain the analytical gels and MS-preparative gels, respectively. For silver staining, the gels were fixed with 40% ethanol and 10% acetic acid overnight, followed by incubated with a buffer solution containing 30% ethanol, 4.1% sodium acetate, 0.2% sodium thiosulfate and 0.125% glutaraldehyde for 30 min. After washing with D. D. H_2_O for three times, the gels were stained with 0.1% silver nitrate solution containing 0.02% formaldehyde for 20 min. Then, after washing with D. D. H_2_O twice, the gels were developed in 2.5% sodium carbonate containing 0.01% formaldehyde, and the reaction was terminated with 1.5% EDTA (disodium salt) followed by thorough rinsing with water. Subsequently, the gels were imaged using a proXPRESS 2D imaging system (PerkinElmer, Waltham, MA, USA) and analyzed with ImageMaster 2D Platinum software 6.0 (GE Healthcare, Fairfield, CT, USA). Only those spots that changed consistently and significantly (more than 2.0-fold) in three replicates were selected for MS analysis.

### 4.6. Protein Identification by MS

For protein identification, spots of interest were selected manually, and tryptic in-gel digestion was subsequently performed [56]. MS analysis was achieved with a 5800 MALDI-TOF/TOF mass spectrometer (AB SIEX, Framingham, MA, USA) [57]. Combined MS and MS/MS spectra were searched against the SwissProt database (Release 2013_1) using Mascot (Matrix Science, London, UK). Search parameters were as follows: taxonomy was limited to Mus musculus; trypsin digestion with only one missed cleavage site was accepted; variable modifications allowed for carboxyamidomethylation of cysteine and oxidation of methionine; 100 ppm for precursor ion tolerance and 0.3 Da for fragment ion tolerance.

### 4.7. Western Blot Analysis

Western blot analysis was carried out using primary antibodies (Abs) against annexin A5 (Boster, Wuhan, China), cofilin-2 (GeneTex, San Antonio, TX, USA), galectin-1, cofilin-1, fructose-bisphosphate aldolase and Cu/Zn-superoxide dismutase (Bioss Biotechnology, Beijing, China), and β-tubulin (Abcam, Cambridge, UK) at optimized dilutions. β-tubulin served as an internal control. Proteins (30 μg/lane) were resolved on 12% SDS-PAGE and transferred onto a PVDF membrane. The blots were incubated overnight at 4 °C with a primary Ab followed by incubation with a horseradish peroxidase (HRP)-conjugated goat anti-rabbit IgG secondary antibody (1:5000, Neobioscience Technology, Shenzhen, China) for 2 h at RT. The blots were then developed using an enhanced chemiluminescence kit (Amersham Pharmacia Biosciences, Buckinghamshire, UK).

### 4.8. Cell Morphology and Actin Cytoskeleton Organization

RAW264.7 cells (2 × 10^5^) were maintained on sterile glass coverslips (Corning Life Sciences Inc., Tewksbury, MA, USA) in 35 × 35 mm^2^ culture dishes with complete medium. After stimulation with 1 mg/mL GOS for 24 h, stimulation with 1 μg/mL LPS alone for 24 h, or pretreatment with GOS for 2 h followed by stimulation with LPS for 22 h, the cells were washed three times with PBS. Then, they were fixed with 4% paraformaldehyde for 15 min at room temperature (RT) and incubated with DAPI (KeyGEN Biotech, Nanjing, China) for 10 min at RT or with TRITC-labelled phalloidin (1:200) (Sigma-Aldrich, St. Louis, MO, USA) in the dark for 20 min at 4 °C for staining of the F-actin fibers. For examination of cell morphology and the actin cytoskeleton observation, fluorescence and dark-field microscopy were performed using an upright Olympus BX51 optical microscope (Olympus Corporation, Tokyo, Japan), and fluorescence and differential interference contrast microscopy were performed using an Olympus FV1000 confocal scanning laser microscope (Olympus Corporation, Tokyo, Japan).

### 4.9. Statistical Analysis

All the experiments were repeated at least three times (*n* ≥ 3), and all types of samples (treated with GOS and untreated) were prepared in triplicate and run in three different 2-DE gels. The data are expressed as the mean ± standard deviation (SD) and were analyzed using the two-tailed Student’s *t*-test to determine any significant differences. *P* values < 0.05 were considered statistically significant.

## 5. Conclusions

AOS, which play important roles in modulating the immune system, are gaining increasing attention as potential biomaterials. Combining the results from our previous studies [16,18] together with those of the present study, we have broadened the understanding of the mechanism by which GOS maintains a proper balance between the pro- and anti-inflammatory immune responses. Differentially expressed proteins extracted from control and GOS-treated RAW264.7 cells were identified by a proteomic study, which were involved in the functional pathways of activation and negative regulation of the NF-κB signaling pathway, pro- and anti-inflammation, pro- and anti-oxidation, cytoskeletal remodeling and cell proliferation in GOS-induced macrophage activation and immunomodulation. GOS is therefore a promising agent that may be utilized by the pharmacological industries to regulate multiple functional pathways and inflammatory responses.
